# Challenges in laser tattoo removal: the impact of titanium dioxide on photodegradation of yellow inks

**DOI:** 10.1007/s00204-025-03989-2

**Published:** 2025-03-06

**Authors:** Batool A. Aljubran, Kirstin E. Ross, Ula Alexander, Claire E. Lenehan

**Affiliations:** College of Science and Engineering, Flinders University, Sturt Rd, Bedford Park, Adelaide, SA 5042 Australia

**Keywords:** Tattoo ink, Laser tattoo removal, Pigment yellow, Titanium dioxide

## Abstract

As tattoos have grown increasingly popular, there has been an increase in their removal. This is commonly achieved using laser treatments. However, certain tattoo inks are resistant to removal using laser methods because of their composition. This includes the removal of yellow pigments and tattoo inks containing titanium dioxide (TiO_2_). This research examined a series of yellow pigments (PY14, PY74, PY65) and tattoo inks, pre- and post-irradiation, with a QS Nd:YAG laser irradiation at 532 nm. The pigments and products were analysed using a range of techniques, including EDX-SEM, DLS, XRD and GC–MS. Results of this study indicate that the presence of TiO_2_ alters the laser degradation process of the pigments studied, with observable changes to particle morphologies, particle size, and evolved volatile products. In addition, some of the degradation products were identified to be potentially harmful to the human body.

**Supplementary Information:**

The online version contains supplementary material available at 10.1007/s00204-025-03989-2.

## Introduction

Tattooing is an increasingly common practise that involves injecting tattoo inks into the skin to create a permanent mark or a visual design (Karadagli et al. 2022). Notably, despite the growing popularity of tattoos, there is also a growing demand for their removal as people experience tattoo regret (Gurnani et al. 2022; Naga and Alster 2017). With the development of improved technology and treatment approaches, tattoo removal processes continue to improve (Brauer et al. 2012; Saedi et al. 2012). Laser tattoo removal is presently the most commonly used approach, often using quality switched neodymium-doped yttrium aluminium garnet (QS Nd:YAG), which seems to be successful due to selective photothermolysis of the chromophores (Anderson and Parrish 1983; Ara et al. 1990; Bauer et al. 2020; Gurnani et al. 2022). The presence of residual tattoo colour is one of the most important indicators of the effectiveness of laser tattoo removal and helps to assess how much pigment remains in the skin, which guides further treatment decisions (Alabdulrazzaq et al. 2015).

Tattoo ink is made up of insoluble pigments suspended in a solvent-based binder along with additives and preservatives that are used to stabilise it and avoid microbiological degradation (Negi et al. 2022). Even when the inks’ ingredients are understood, there are dangers associated with laser removal. This includes the creation of potentially dangerous particles, both from the pigment itself, and the medium (i.e., the two components of a tattoo ink) (Ara et al. 1990; Bauer et al. 2020; Engel et al. 2010; Ma et al. 2017; Moseman et al. 2024).

Moreover, it has been recorded that the ingredients and colours of tattoo inks contribute to the effectiveness of laser removal procedures, potentially influencing the number of treatment sessions (Engel et al. 2010; Hauri and Hohl 2015). For example, dark-coloured tattoo inks can be efficiently removed with a limited number of treatments using laser such as picosecond alexandrite laser (Saedi et al. 2012). Light colours such as yellow and white pigments, on the other hand, have been found to be difficult to completely eliminate, since the existing accessible laser wavelengths are not well absorbed by the pigments (Beute et al. 2008; Kim et al. 2006). In addition, in vitro investigations showed that the wavelength of the yellow ink’s absorption peak (440 nm, 470 nm, and 485 nm) does not match the current wavelengths that are available in QS laser systems (Alabdulrazzaq et al. 2015; Beute et al. 2008; Saedi et al. 2012). One study has indicated that using a QS Nd:YAG laser with a nanosecond pulse duration can been effective to remove yellow tattoo inks pigments. However, the complete removal of these yellow inks remains challenging and uneven (Alabdulrazzaq et al. 2015; Ferguson and August 1996). Yellow ink had traditionally been a recalcitrant colour to remove using the QS laser instrument, until the development of the picosecond lasers, which effectively remove this ink colour (Alabdulrazzaq et al. 2015; Naga and Alster 2017).

Certain tattoos might be resistant to treatment not only due to poor absorption of the laser’s radiation by the tattoo pigment, but also due to oxidative–reductive alterations in some metals when excited by laser light. One example is the phenomenon where tattoos containing iron oxides tend to darken when treated with high-powered laser therapy (Anderson et al. 1993; Butterfield 2022; Lehmann and Pierchalla 1988; Timko et al. 2001). Titanium dioxide (TiO_2_) is commonly used to enhance the brightness of various coloured tattoo inks, such as blue, yellow, green, and purple (Bäumler et al. 2000; Ross et al. 2001). The presence of TiO_2_ has been widely reported to make tattoo removal more difficult, and in most clinics, laser removal of tattoo ink based on TiO_2_ is not achievable (Ho and Goh 2015; Kim et al. 2006; Timko et al. 2001). Tattoos containing TiO_2_ have been stated to act strangely, with the TiO_2_ changing colour after the treatment from white to a “filthy” green, dark grey, blue, or pale purple, or not responding to laser light at all (Lichnyi et al. 2021). Prior research has indicated that TiO_2_ can undergo a transformation from white to black under laser treatment (Chen et al. 2015). Others have reported that TiO_2_ is an exceptionally strong substance that is difficult to break down without several laser removal treatments (Darby 2013). This is problematic as, in terms of laser removal of TiO_2_-containing tattoos, it has been noted that “repeated visits usually cause scarring of the skin” (Lichnyi et al. 2021).

It has been proposed that the reduction of white and other titanium-containing inks causes the blackened colour of irradiated tattoos (Ross et al. 2001), and showed a similar effect for a titanium-enriched sunscreen exposed to radiation (Ross et al. 2001). This was attributed to the reduction of Ti^4+^ to Ti^3+^, as demonstrated by Torimoto and co-workers (Ross et al. 2001; Torimoto et al. 1996). Because of the intense nature of the nanosecond pulses typically employed in laser therapy, even slight absorption of other wavelengths can lead to a darkening response, primarily driven by the reduction of TiO_2_ within the ink (Torimoto et al. 1996). The impact of TiO_2_ quantity on the extent of darkening remains uncertain. As many yellow tattoo inks commonly include TiO_2_ to enhance their brightness, this is also likely contributing to the phenomenon where yellow tattoo inks turned black after laser exposure (Varma et al. 2002). This is consistent with a report by Kim and colleagues (2006), who showed that blue tattoo ink containing TiO_2_ exhibited resistance to Nd:YAG laser therapy *(*Kim et al. 2006).

In addition to colour changes, there is limited knowledge on the effects of laser treatment on ink particle size and morphology. Murphy (2018) suggested that ink may be re-aggregated and ejected at high speeds, but no analytical data were offered to back up these claims (Murphy 2018). Reaggregation of the ink particles would make it potentially increasingly difficult to remove tattoos. It is vital to understand how laser treatments affect ink particles, since size and morphological changes in treated inks are likely to occur throughout the removal process.

This research investigated the influence of TiO_2_ on the laser degradation of pigment yellow, a typical component of tattoo inks, to determine the effect on resulting colour, particle size, and morphology. The study purposefully used a simple model, combining the pigment with only TiO_2_ and exposing it to laser irradiation. This deliberate exclusion of other components mimicked the controlled conditions relevant to laser tattoo removal, specifically aiming to reduce the complexity introduced by other skin components during the process. The study examined yellow pigments, tattoo inks, and the degradation products produced when these samples are treated with high laser intensities. A comprehensive array of instrumental techniques, including GC–MS, SEM & EDX, XRD, and DLS, were employed for the characterisation of both unirradiated and irradiated samples. This multidimensional approach facilitated a thorough understanding of the chemical and morphological changes occurring in the studied systems, shedding light on the potential applications and implications of TiO_2_ in laser-assisted tattoo removal.

## Method

### Material and instruments

Lemon Yellow (LY), Golden Yellow (GY), Golden Rod (GR), and Bright Orange (BO) Intenze® brand inks were purchased from Tattoo Direct, Victoria, Australia. Pigment yellow 14 (PY14) (C.I. 21,095) (97%), pigment yellow 65 (PY65) (C.I. 11,740) (98%), pigment yellow 74 (PY74) (C.I. 11,741) (tech), and TiO_2_ (C.I. 77,891) were purchased from AK Scientific, Union City, California. Methylene chloride (99.9%) was purchased from RCI Labscan, Australia. Methanol (LC MS grade) was purchased from Honeywell, Australia. All chemicals used in this investigation, except the tattoo inks themselves, were used without any further purification. The reported ingredients of tattoo inks used in this study according to the manufacturer’s label and the safety data sheets are shown in Table SI1.

### Sample preparation and laser experiment setup

**Fig. 1:**
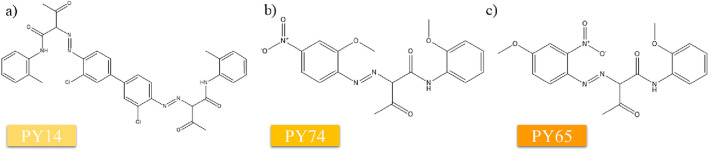
A schematic representation of the chemical structures of **a** PY14, **b** PY74, and **c** PY65

The general method used in this research is presented in Fig. SI1 and chemical structure of reference pigments is shown in Fig. 1a–c. Tattoo inks were pipetted onto microscope slides and dried in open air at ambient temperature for 48 h prior to irradiation and/or characterisation. Dried inks were scraped from the slide and placed in a GC vial prior to laser treatment. The pigment-TiO_2_ samples were made by mixing various pigments (PY14, PY74, PY65) in separate GC glass vials with different particle sizes (300 and 500 nm) of TiO_2_ (rutile form) with a 50:50 w/w % ratio.

Dried tattoo inks and a mixture of PY14, PY74, and PY65 with TiO_2_ were irradiated using a QS Nd:YAG laser at 532 nm. Each sample was exposed to 20 laser pulses (each with a duration of 6 ns) over a period of around 1–2 min. The laser pulses were 157 mJ/pulse over an area of 2 mm diameter (i.e., a fluence of 5 J/cm^2^). As a negative control, PY14, PY65, PY74, and TiO_2_ were irradiated individually. The photodegradation products were analysed using a headspace GC–MS to identify volatile fragments. In addition, SEM, DLS, and XRD were used to study the changes in crystal structure, particle shape, and size following laser irradiation.

### Instrumental analysis

Headspace gas chromatography–mass spectroscopy (GC–MS) analysis was carried out using an Agilent Technologies 7890A GC system with a 5975C inert XL EI/CI MSD Triple Axis Detector and a 7693 sampler. The apparatus was fitted with an Agilent Technologies HP-5MS 5% Phenyl Methyl Siloxane column (29.4 m × 250 µm × 0.25 µm) with a He_2_ mobile phase at a flow rate of 3.5 mL/min. The headspace injection volume was 5 µL. The GC was operated in isocratic mode with an oven temperature of 40 °C and an inlet temperature of 180 °C throughout the analysis. The MS ion source and quadrupole temperatures were set at 230 °C and 150 °C, respectively. All m/z values between 40 and 500 were taken in scan mode. The presence of volatile hazardous chemicals in the irradiated pigments and inks or the eluted compounds was identified by comparison of the mass spectra using the NIST database and comparison to literature (Bauer et al. 2020; Cecchetti et al. 2022; Hering 2022; Hering et al. 2018; Schreiver et al. 2016, 2015). To help determine the identity of the organic volatile compounds, the retention index was measured using the Kovats approach from the headspace GC–MS analysis of stander alkanes (C5–C9) (Agustia et al. 2022; Bianchi et al. 2007; Boegelsack et al. 2021; Zhao et al. 2022).

Scanning electron microscopy (SEM) was undertaken with an FEI F50 inspect system equipped with an Octane Pro energy-dispersive X-ray (EDX) detection system. Pigment samples were prepared by directly spreading pigment powder onto sticky carbon tabs. The working distance was 10 mm, and the acceleration voltage was10 kV. PY14, PY74, GR, and GY inks were coated with platinum with a thickness of about 2 nm to increase their electrical conductivity.

Dynamic light scattering (DLS) measurements were achieved using a Malvern Nano Zeta Sizer apparatus equipped with a 5 mW HeNe laser and a Peltier temperature control system. Backscattering detection at an angle of 173° was used to determine the hydrodynamic size and size distribution of pigments, inks, and TiO_2_ samples. This configuration is less vulnerable to multiple scattering effects and dust than the 90° geometry. Measurements were taken at 20 °C and were repeated three times. Before DLS measurements, all dispersions were prepared by dilution of the pigments with deionized water or methanol, followed by 30 min of sonication at 40 kHz, at a ratio of 0.1 mg pigment to 1 mL of solvent.

X-ray diffraction (XRD) was recorded for pigment samples and dried tattoo inks. Data were collected using a Bruker Advanced D8 diffractometer with Co Kα (*λ* = 1.7889 Å, 2*θ* = 10–90°, time per step = 0.5 s). All samples were ground to a fine powder with a mortar and pestle before being loaded onto an XRD sample stage.

## Results and discussion

PY14, TiO_2_, PY14-TiO_2_, and dried ink samples were irradiated with a 532 nm QS Nd:YAG laser as outlined in the “Sample preparation and laser experiment setup” methods. Visibly, the pigment and ink samples were observed to change colour from yellow (pre-exposure) to a brownish green (post-irradiation) (Fig. SI2). The TiO_2_ samples were observed to turn slightly grey after irradiation. This contrasts with prior reports that have indicated that TiO_2_ turned black post-irradiation (Chen et al. 2015). This is due to that this research used 532 nm laser light, however, TiO_2_ does not absorb radiation at this wavelength. Samples were characterised pre- and post-irradiation using GC–MS, SEM, DLS, and XRD to examine changes in sample chemistry, composition, morphology, and particle size. Results from these analyses are discussed below.

### GC–MS

**Fig. 2:**
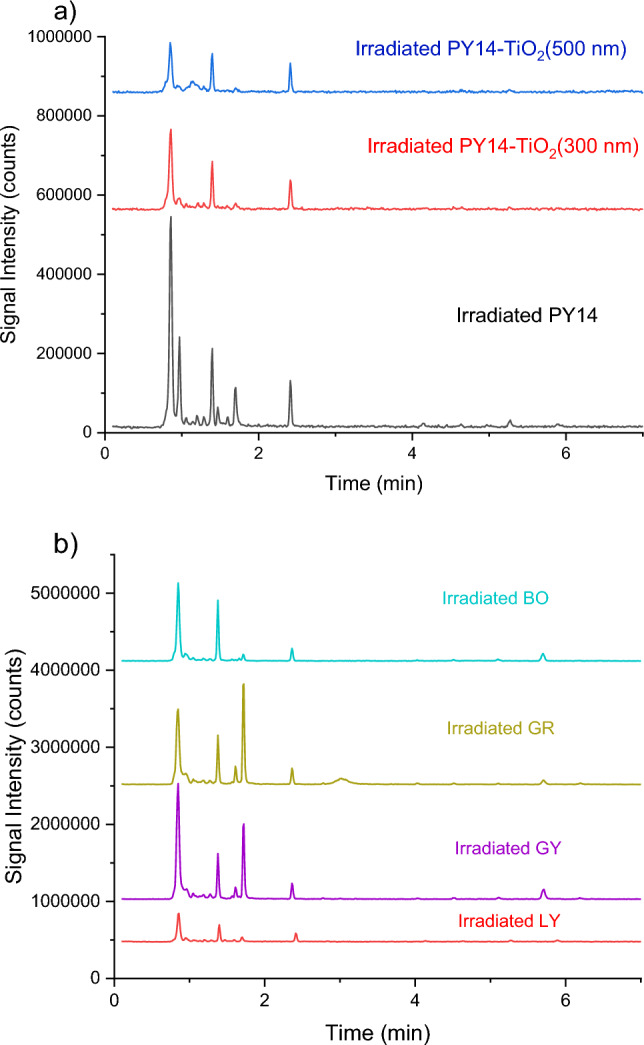
GC–MS chromatogram of irradiated **a** PY14, PY14–TiO_2_ and **b** dried LY, GY, GR, and BO tattoo inks

Headspace GC–MS was carried out to identify volatile breakdown products resulting from the laser irradiation of reference pigments, pigment–TiO_2_ mixtures and inks. Figure 2a shows the GC–MS chromatograms of irradiated PY14, and two irradiated PY14-TiO_2_ mixtures with different TiO_2_ particle sizes. The GC–MS chromatograms of tattoo inks post-irradiation (LY, GY, GR, BO) are shown in Fig. 2b. The GC–MS chromatograms of the irradiated empty vial, unirradiated TiO_2_, unirradiated PY14, and unirradiated inks did not exhibit any peaks (see supporting information Fig. SI3), confirming that the volatile compounds resulted from the irradiation process. Table 1 provides details on fragmentation products identified in the samples post-laser treatment including retention time and primary mass losses for each component. Notably, 1,3-butadiyne benzene and toluene were present across all samples. In addition, dried inks produced 2-propenoic acid-ethyl ester, methyl methacrylate, and styrene, whilst PY14 produced 3-butenyl methacrylate, and a peak at 1.7 min that was tentatively assigned as either *N,N'*-dimethyl-1,2-bis(aminooxy)ethane, or methyl 1-dideutrero-2-propenyl ether. Neither of these are readily attributed to fragmentation of the pigment or rearrangement of volatile fragments, and further work is required to confirm the identity of this peak. Both PY74 and PY65 generated 2-propenenitrile, whereas benzyl alcohol was formed from PY65.

**Table 1:** The volatile fragmentation products released during laser irradiation of pigments, inks, and the retention times, the retention index, and major mass losses

Retention time (min)	Compound	Main fragments (m/z)	Observed										Retention index
			PY14	PY74	PY65	TiO_2_			LY	GY	GR	BO	
						PY14	PY74	PY65					
0.849	1,3-Butadiyne	50, 49, 48	✓	✓	✓	✓	✓	✓	✓	✓	✓	✓	–
0.920	3-Butenyl methacrylate	69, 54, 51	✓	–	–	–	–	–	–	–	–	–	594.29
0.953	2-Propenenitrile	53	–	✓	✓	–	✓	✓	✓	–	–	–	–
1.144	Benzamide/benzonitrile	103, 76, 50	–	–	–	✓	–	–	–	–	–	–	631.37
1.397	Benzene	78, 50	✓	✓	✓	✓	✓	✓	✓	✓	✓	✓	667.25
1.635	2-Propenoic acid-ethyl ester	99, 55	–	–	–	–	–	–	–	✓	✓	-	768.33
1.70	*N, N’*- dimethyl-1,2-bis(aminooxy)ethane	120, 74	*	–	–	–	–	–	*	–	–	–	–
1.70	Methyl 1-dideutrero-2-propenyl ether	74	*	–	–	–	–	–	*	–	–	–	–
1.722	Methyl methacrylate	100, 69	–	–	–	–	–	–	–	✓	✓	–	–
2.412	Toluene	91, 65, 51	✓	✓	✓	✓	✓	✓	✓	✓	✓	✓	770.86
5.701	Styrene	104, 78, 51	–	–	–	–	–	–	✓	✓	✓	✓	891.27
7.208	Benzyl alcohol	108, 78	–	–	✓	–	–	–	–	–	–	–	931.74

*The volatile fragment was tentatively identified

The presence of benzene, toluene, and styrene is consistent with previous studies on the laser irradiation of suspensions containing pigment blue 15, pigment green 7 and 36, pigment yellow 138, pigment orange 13, pigment violet 19, as well as pigments red 170 and 245 (Bauer et al. 2021; Cecchetti et al. 2022; Hering et al. 2018; Schreiver et al. 2016, 2015). 2-propenenitrile was reported to be formed by thermal chain scissions of the binders, solvents, and additives of Edding felt-tip pen inks (Germinario et al. 2018). The formation of the remaining compounds (1,3-butadiene, 2-propenoic acid-ethyl ester, methyl methacrylate, 3-butenyl methacrylate, benzyl alcohol) from the dried inks and pigments has, to our knowledge, not previously been reported. These products could potentially be attributed to the impurities present in the pigments during the manufacturing process or other ingredients in the tattoo inks which could later degrade under laser irradiation. Additionally, the intense energy from the laser might induce rearrangement or fragmentation of the pigment molecules themselves, leading to the formation of new compounds not originally present in the pigment. The variation in the products formed implies an influence of the ink matrix on fragmentation during laser irradiation, which is changed when the ink is partially removed from the vehicle layer and the pigment aggregated.

When the GC–MS chromatograms from irradiated dried tattoo inks were compared to that of the irradiated organic reference pigments, the inks exhibited higher intensity peaks. It is hypothesised that the particles in the inks have a greater specific surface area compared to agglomerated pure pigments, leading to altered response rates to the laser light.

The number of peaks observed in the GC–MS chromatogram from the irradiated PY14–TiO_2_ mixtures was decreased when compared to irradiated PY14. Fragments observed in irradiated PY14 at 0.92, 1.458, and 1.702 min were absent from the GC–MS chromatograms of the PY14–TiO_2_ mixtures. Additionally, a reduction in peak intensity was observed in the chromatograms of irradiated PY14-TiO_2_ mixtures when compared to the irradiated PY14. This appears to be dependent on the TiO_2_ particle size, with mixtures of PY14 and 500 nm TiO_2_ having lower intensities than the mixture containing 300 nm TiO_2_. In addition to these changes, an additional peak with low intensity at 1.144 min was detected as a photodegradation product from irradiated PY14–TiO_2_ but not in the irradiated PY14. Based on the MS data, this could be attributed to either benzamide or benzonitrile. Both are potential degradation products based on the structure. Benzonitrile was reported as a laser decomposition product from PO13 (Hering et al. 2018). Similar changes in the GC–MS chromatograms were observed for PY75 and PY65 when irradiated in the presence of TiO_2_ (Fig. SI4). These results are consistent with previous research which stated that TiO_2_ alters the photodecomposition of PO13 (Schreiver 2018), and are likely to be attributable to the presence of TiO_2_ and its capacity to absorb or reflect laser light, which changes how laser light interacts with these yellow pigments. The presence of TiO_2_ may reduce the energy of light available to breakdown the PY14, hence reducing the intensity of the volatile fragments in the GC–MS peak decreases. Additionally, this might result in novel interactions between volatile compounds produced during laser irradiation and the rearrangement of the volatile fragments, which would produce new molecules.

The effect of TiO_2_ on laser degradation of inks was verified by comparing the GC chromatograms of GR and BO inks (Fig. 2b). These inks have a very similar ingredient list, only differing in that GR does not contain TiO_2,_ whilst BO is listed as containing TiO_2_ (25%, according to the label). In BO ink, the intensities of the peaks at 1.635 and 1.722 min from 2-propenoic acid-ethyl ester and methyl methacrylate were reduced significantly. Furthermore, the signal intensity of additional fragments was lower in BO ink than in GR ink. This result provides further evidence to suggest that the presence of TiO_2_ alters the way the inks interact with laser light and reduces amount of fragmenting.

### SEM

**Fig. 3:**
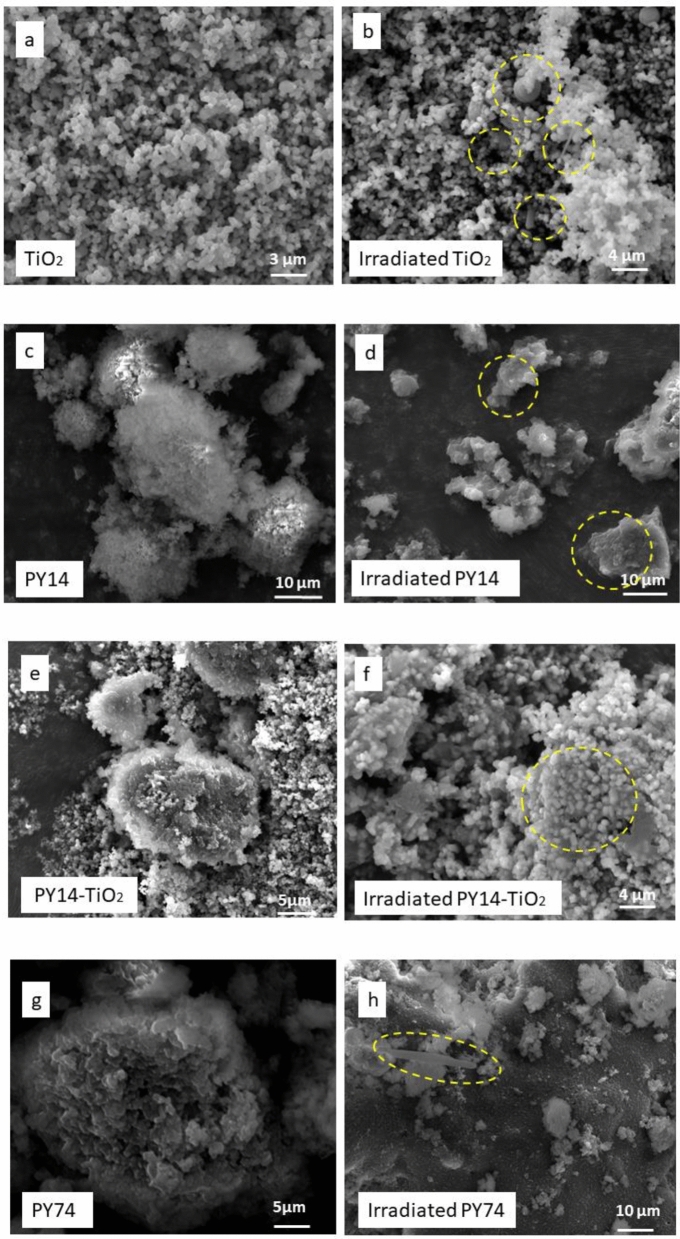

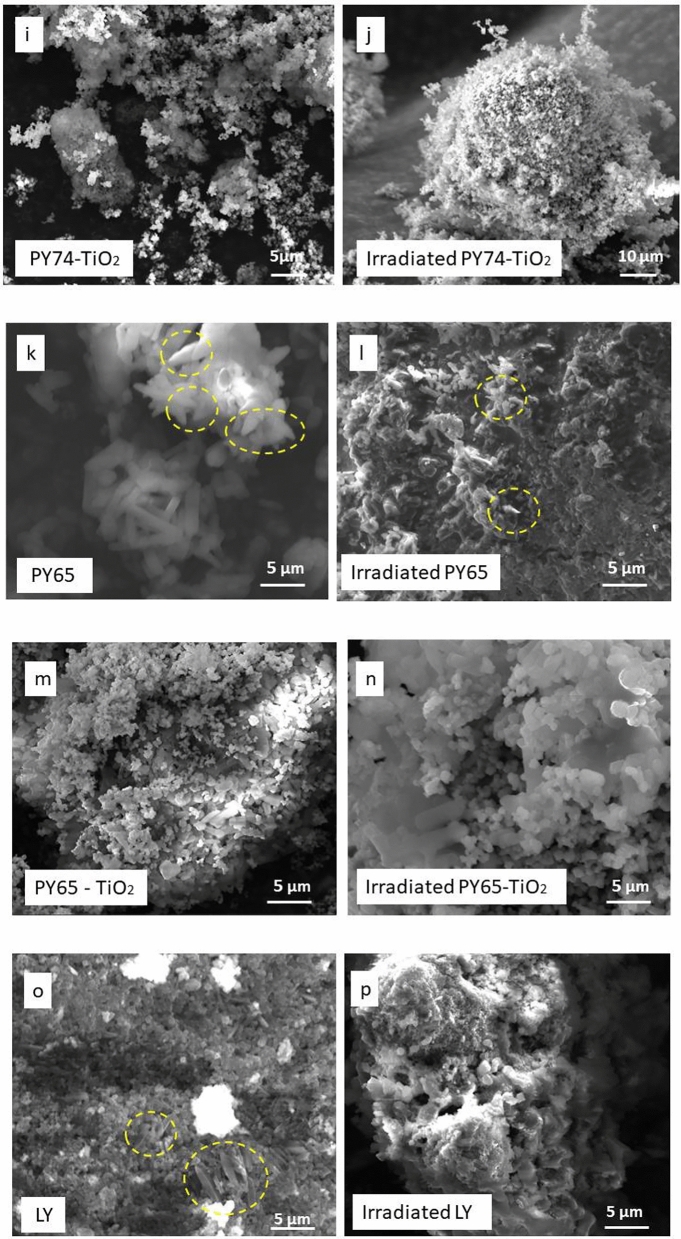

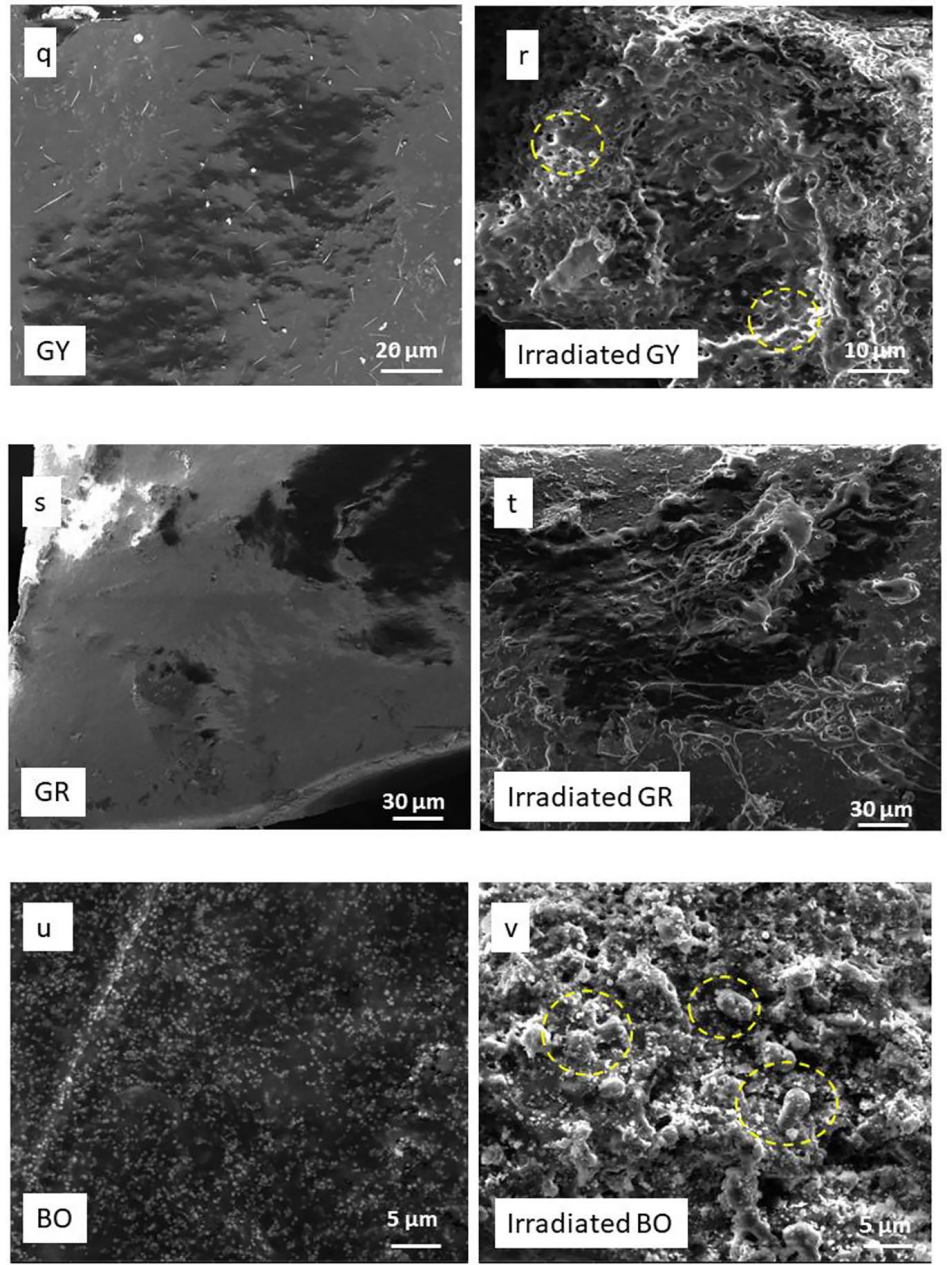
SEM images at different magnifications of unirradiated and irradiated PY14, PY74, PY65, TiO_2_, mixtures of pigments with TiO_2_, and dried tattoo ink. This shows the change in the morphology of pigments and inks after laser irradiation. TiO_2_ aggregates around pigments

PY14, TiO_2_, PY14–TiO_2_ mixtures and tattoo inks were examined pre- and post-laser irradiation via SEM to ascertain any morphological changes (Fig. 3). As can be seen, prior to irradiation, the SEM images of TiO_2_ showed cuboid rectangular shapes with edges and corners, typical of TiO_2_, and consistent with previous study (Zuñiga-Ibarra et al. 2019) (Fig. 3a). Irradiated rutile TiO_2_ nanoparticles revealed a change in morphology when compared to unirradiated TiO_2_ (Fig. 3b). There are some agglomerated, larger spherical nanoparticles, long rods as well as tiny nanoparticles in this sample (identified in the Fig. 3b). Rather than simply breaking apart the TiO_2_, the laser irradiation appears to have a heating and melting impact on TiO_2_ particles. Laser-induced fragmentation of larger particles leads to the formation of smaller particles. As the irradiation proceeded, the smaller nanoparticles remaining might be heated and melted at high temperatures above 1000 °C (Adatto 2004; Kirby 2013). These shape alterations could be caused by laser photon energy absorption (including non-linear absorption and heating or melting of the nanoparticles) causing alterations in their phase and crystalline structure. Unirradiated PY14 consisted of finely aggregated powders; however, post-irradiation the PY14 sample was reorganised into thinly layered smooth sheets of 2–6 µm in length (Fig. 3c, d). Interestingly, the SEM images of the irradiated PY14-TiO_2_ show that the particles had agglomerated, with the TiO_2_ appearing to be “glued” onto the PY14 surface as smaller, irregularly shaped particles (Fig. 3e, f). Additionally, it appears TiO_2_ forms clusters around PY14 particles. This may play a role in preventing the fragmentation of PY14 into smaller particles and contribute to the decreased signals in the GC–MS which are presented in Fig. 2a.

Similar results demonstrating the effect of the photoactivity of TiO_2_ were also observed in the SEM images of irradiated PY74–TiO_2_ and PY65–TiO_2_. In comparison with the unirradiated mixture of PY74–TiO_2_ (Fig. 3i), the irradiated mixture demonstrated that TiO_2_ agglomerated around PY74 particles as a coating layer (Fig. 3j). It is interesting to note that PY65 exhibits unique micro-morphologies including cubes, rods, spheres, and undefined-shaped microcrystals (Fig. 3k). The smoothness was also seen in the irradiated PY65–TiO_2_, with more intense melting and a vacuous look with small floating particles in between (Fig. 3n). In addition, SEM of the irradiated PY65–TiO_2_ showed TiO_2_ particle adhesion on the surface of PY65. The adhesion of TiO_2_ to the pigment's surface supports the hypothesis of the photoactivity of white pigment upon exposure to laser light, causing the pigments to heat up and agglomerate with these yellow pigments. EDX was carried out to verify between yellow pigments and TiO_2_ particles (Fig. SI5).

Tattoo inks are a combination of pigments and other components; therefore, their morphologies vary depending on their content, and various particle shapes and sizes show up in the same sample (Høgsberg et al. 2011). Smoothness of surface was the shared feature between GY, GR, and BO inks (Fig. 3q, s, u). However, SEM analyses are performed on dried ink samples, and the behaviour of the particles in solution may differ, i.e., the removal of solvent may increase aggregation and agglomeration in the original inks (Bocca et al. 2017). After irradiation, damage to the smoothness of the dried inks’ surface was observed. Pits formed that extended to the entire surface, with the remaining intact surface creating islands between them and the structure became more porous. Following laser irradiation, fibre structures sized in the micrometre range and arranged in a random structure of LY ink were melted (Fig. 3o, p). Other characteristics of the irradiated tattoo ink sample include coalescing blocks and punctured regions with wide holes (the formation of cavities and pores). Moreover, it has been reported that Cu (found in PB15) is a promoter and one of the ingredients in LY ink; therefore, particle agglomeration may occur (Mingmongkol et al. 2021). In this work, irradiated BO ink had a different morphology compared with the GY and GR inks (Fig. 3r, t, v). SEM images of irradiated BO inks revealed greater heterogeneity, with spheres and tiny particles aggregated on the ink's surface. An EDX investigation of the irradiated sample revealed that the surface alterations were due to a large amount of TiO_2_ appearing on the surface (Fig. SI6). This is likely due to a higher concentration of TiO_2_ in the ink, when compared with the GR and GY ink.

The laser treatment indicates that the shape and size alteration of inks and pigments particles occurred by particle melting. If this is the case, the measured laser fluence threshold for the transition should correspond to the fluence at which the particles’ temperature surpasses the melting point of pigment (Zhigilei and Garrison 1998). According to the literature, the optical properties of a pigment, specifically its colour and hiding power, are determined by the shape and size of its particles (Gueli et al. 2017). Therefore, the darkening of inks and yellow pigments (Fig. SI2) could be a result of the alterations of the size and the morphology after irradiation with laser. The mechanism during irradiation that results in morphological changes is most likely connected to the temperature at the focussing location. It has been assumed that all irradiated pigments are broken into smaller particles, and as a result, laser removal is generally a successful method in the tattoo removal process. However, in the presence of TiO_2_, the particle size appears to increase, making the removal process difficult. The aggregation phenomena are presumably the combined effects of heating and beam penetration, impacting both the pigment and the carrier in tattoo inks at the same time.

### DLS

**Fig. 4:**
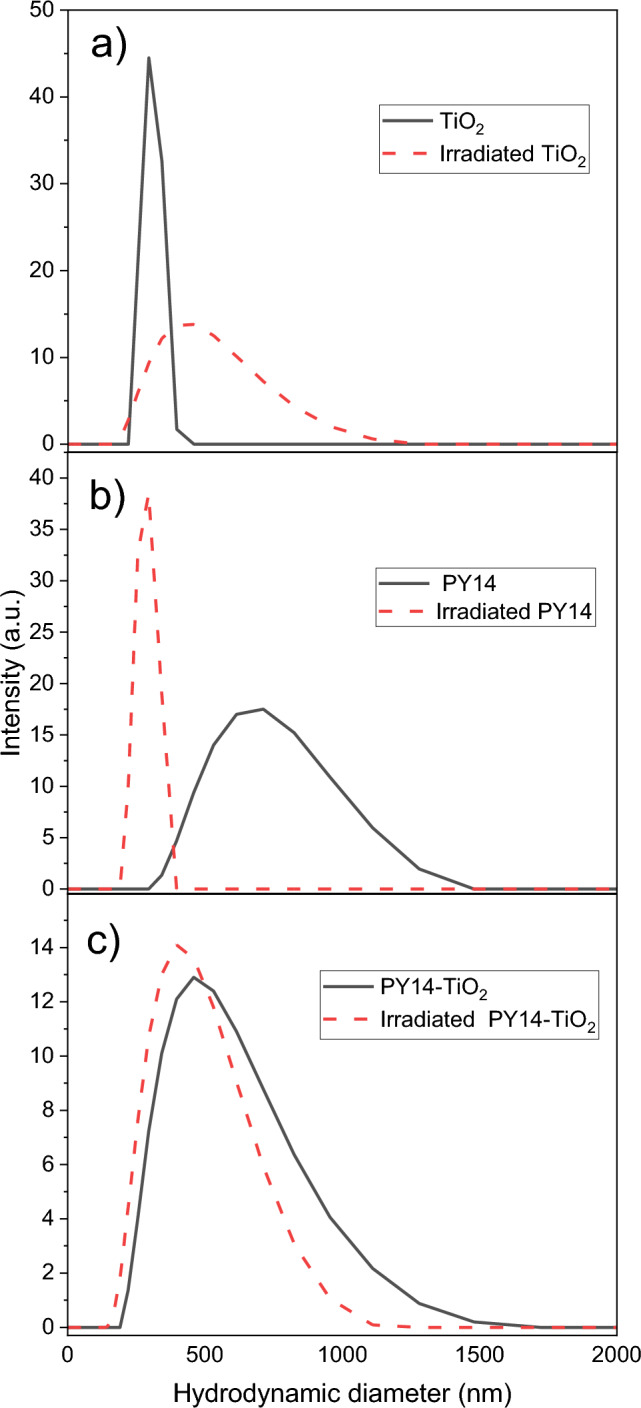
DLS data of unirradiated and irradiated **a** TiO_2_, **b** PY14, and **c** PY14-TiO_2_. Comparison of the particle size of irradiated pigments with and without TiO_2_, showing the particle size of irradiated pure pigments is smaller than irradiated PY-TiO_2_

**Fig. 5:**
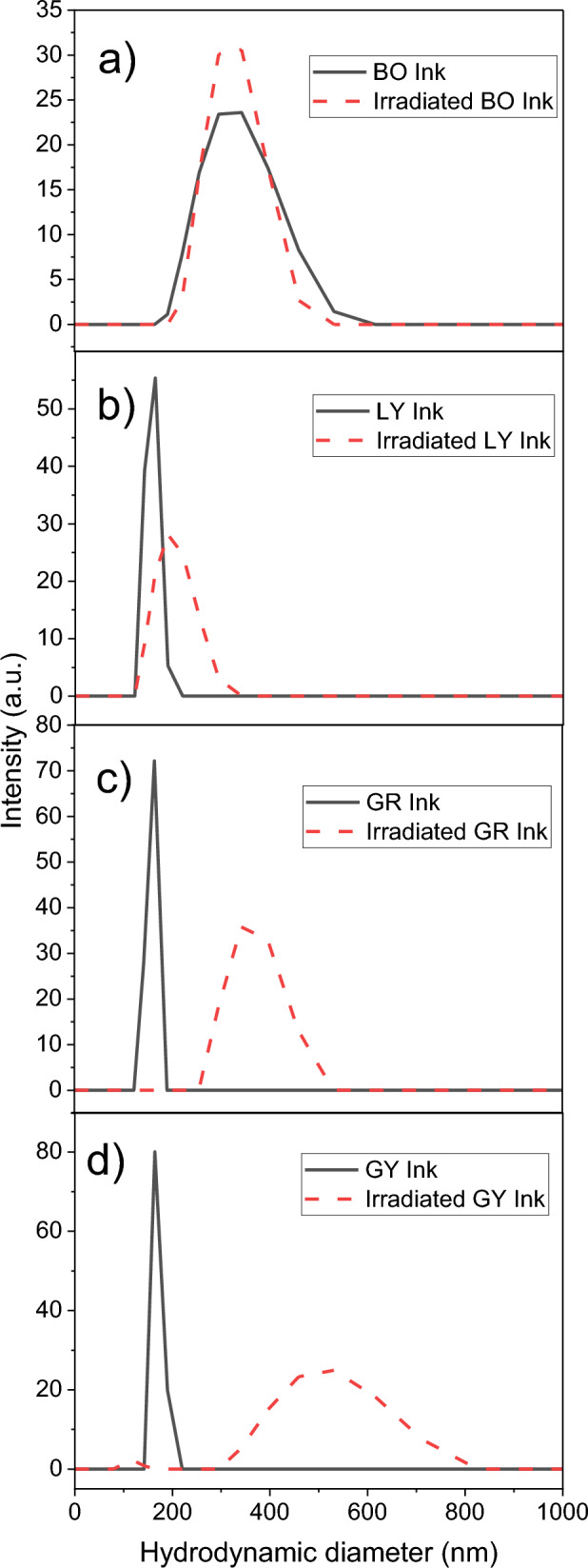
DLS data of unirradiated and irradiated **a** BO, **b** LY, **c** GR, and **d** GY inks

The effect of laser irradiation on particle size was explored using DLS. The nanosized diameter of suspensions of unirradiated and irradiated PY14, PY14–TiO_2_, and ink particles was examined (Figs. 4, 5; Table S12). As can be seen from Fig. 4, the DLS measurements provides the overall average size and shows that 300 nm TiO_2_ agglomerated to a large particle size of around 473 ± 7 nm after laser irradiation. However, PY14 particles fragmented from 705 ± 39 nm (unirradiated) into smaller particles with a diameter of around 301 ± 20 nm when irradiated. For TiO_2_ pigment mixtures, the irradiated mixtures had a larger size of around 461 ± 29 nm when compared to irradiated PY14 and were similar in size to the irradiated TiO_2_. This research indicates that the TiO_2_ plays a major role in the overall final particle size of irradiated PY14–TiO_2_ mixtures. It is hypothesised that this phenomenon is linked to reduced absorption of laser light by PY14 in the presence of TiO_2_ which hinders fragmentation into smaller particles during treatment. Furthermore, considering the elevated local temperatures greater than 1000 °C during laser removal (Adatto 2004; Kirby 2013), it is more probable that the particles melt and form larger particles.

These findings align with SEM results showing increased particle sizes post-irradiation forming spherical shapes. Notably, whilst SEM identified higher particle sizes than DLS measurements, this discrepancy may be attributed to differences arising from agglomerate dispersion within the DLS solution.

A similar trend was also observed for PY65 and PY74. Laser irradiation of PY65–TiO_2_ resulted in a size shift of the particles from 272 ± 16 nm (irradiated PY65) to 455 ± 13 nm. Similarly, DLS analysis showed that irradiated PY74–TiO_2_ exhibited a significant change in hydrodynamic diameter from 165 ± 21 nm (irradiated PY74) to 334 ± 14 nm, attributed to varying amounts of TiO_2_ present (Fig. SI7).

Prior to irradiation, LY, GY, and GR have similar particle sizes of around 164 ± 35 nm, with BO having a larger average particle size of around 329 ± 20 nm. The larger particle size of BO is likely due to the large quantity of TiO_2_ compared with the remaining inks which had no TiO_2_ (GR) and smaller quantities (LY and GY) (Table SI1). All of the inks had larger particle sizes after irradiation; however, the magnitude of the change varied. Interestingly, BO ink showed only a small increase in the ink’s particle size after laser treatment, which could support the hypothesis that the presence of TiO_2_ in high quantities limits the effect of the laser on particle breakdown and resulted in aggregates of this tattoo ink. A larger increase in particle size was observed for GY and GR inks following laser irradiation. Further suggesting that the small amount of TiO_2_ (1–10%) has less effect on the particle size. It would be expected that if the particle size from the ink was just dependent on the pigment, a smaller particle size would result after irradiation. However, the inks comprise both pigment and TiO_2_ along with a number of other vehicles. These vehicles may also be playing a role.

The DLS data support the SEM observations, particularly concerning agglomerates, even though large agglomeration structures may exceed the detection range of DLS. For irradiated pigments and tattoo inks, both SEM and DLS indicate heterogeneous structures with diameters ranging from 250 to 800 nm. In the case of the DLS technique, as per Mie's theory, where scattering intensity follows a power-law relationship with radius size, it is plausible to infer that larger aggregates could potentially mask smaller structures that might be less than 100 nm in size (Tomaszewska et al. 2013).

### XRD

**Fig. 6:**
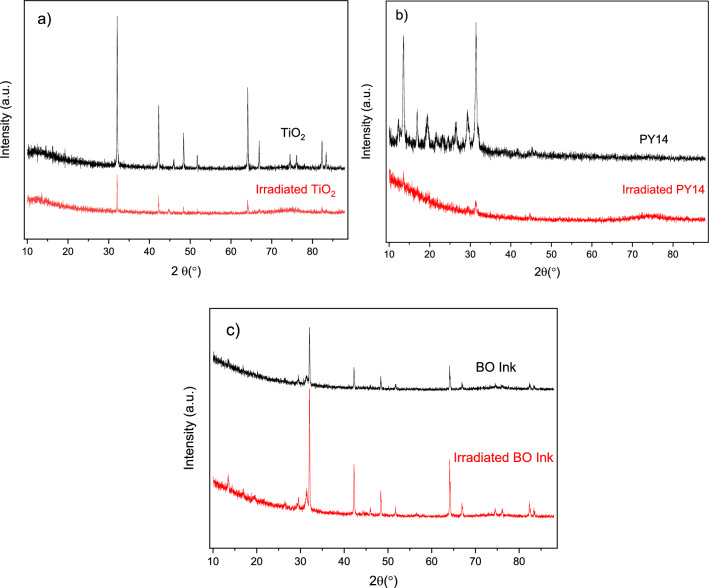
XRD analysis of unirradiated and irradiated **a** TiO_2_, **b** PY14, **c** BO Ink. TiO_2_ and PY14 present how the crystal structure change after laser irradiation. BO ink surface analysis shows high-intensity peaks of TiO_2_ after laser irradiation

XRD analysis was conducted on both unirradiated and irradiated pigments and inks to explore the potential relationship between pigment darkening, molecular composition alterations, binding medium degradation, and changes in phase structures. The XRD analysis of irradiated TiO_2_ and PY14 indicated a rearrangement of the crystal structure, as evidenced by decreased intensity of diffraction peaks (Fig. 6a, b). LY, GY, and GR inks behaved similarly (Fig. SI8). In contrast, the XRD data of BO ink (Fig. 6c) showed more pronounced high-intensity peaks after laser irradiation. This increase in intensity is likely due to TiO_2_’s arrangement towards the surface of the BO ink after exposure to 532 nm laser light, as shown in the SEM images (Fig. 3v). The SEM images, together with the XRD results, suggest that TiO_2_ undergoes photoactivity or photoreaction upon laser light exposure, potentially indicating a structural transformation from rutile to anatase (An et al. 2023; Chen et al. 2015).

The findings from this study support the hypothesis that pulsed-laser irradiation induces changes in the crystalline structure and phase of the pigments. Chemical or photochemical redox processes may contribute to colour changes in inorganic and metallo-organic pigments. Given the high local temperatures (> 1000 °C) generated during laser removal (Adatto 2004; Kirby 2013), these reactions are more likely to occur, which could explain the common darkening of tattoos post-treatment. Additionally, local colour changes might result from redox reactions involving photocatalytic TiO_2_ and copper phthalocyanine pigments, such as Blue 15 and Green 7. Organic pigments could decompose into colourless or coloured fragments through chemical and/or photochemical reactions, which in some cases may pose health risks (Butterfield 2022; Ross et al. 2001). The most compelling evidence of the thermal effect is the observed colour shift and pigment darkening following laser irradiation, likely driven by heat-induced changes in the crystal lattice structure of each pigment.

## Potential health concerns

This study raises significant health concerns related to the in vivo formation of potentially toxic molecules and aggregates upon the laser irradiation of pigments and tattoo inks. GC–MS analysis identified several volatile fragments, which all have potential health effects as outlined in their safety data sheets obtained from Chemwatch (ChemWatch). These health hazards are summarised in the supporting information (Table SI3). For example, compounds, such as 2-propenenitrile, benzene, methyl methacrylate, toluene, and styrene, are known to induce toxicity/harm (H311, H312), irritation (H315), and allergic reactions (H317) upon contact with the skin. Furthermore, benzene is classified as carcinogenic (H350), and methyl methacrylate is recognised for causing severe eye damage (H319) and is also a skin irritant, whilst toluene may harm the unborn child (H361d) and can cause organ damage with prolonged or repeated exposure (H373). These hazardous chemicals are typically encountered through inhalation or skin contact. This research indicates that these toxic compounds may be formed within the body during laser treatment, adding a new route of exposure. There is limited understanding of the risks from this new route of exposure when compared to exposure through skin and respiratory routes. In any case, there is concern that in vivo generation of these compounds could result in more significant health effects. Importantly, during laser irradiation, the compounds are likely concentrated at the treatment site, which could potentially lead to significant localised damage. Following this, they may be distributed through the body through the blood and lymphatic systems. The level of hazard will depend on their likelihood to be excreted from or accumulate within the body. In addition, results from this study illustrates that the presence of TiO_2_ makes laser pigment removal more challenging; thus, additional sessions may be required. This would result in repeated exposure to these volatile hazardous fragments, but at a lower dose per session.

In addition to the formation of volatile compounds, DLS results shows that laser irradiation of PY14, PY74, and PY65 led to the formation of smaller nanoparticles. These substances could be redistributed in the body and cause harmful or even carcinogenic responses (Egbuna et al. 2021; Kumar and Dhawan 2013). SEM of the irradiated pigments also showed that the produced morphologies were highly diverse, with some of them potentially hazardous when in touch with or expelled from the skin. For example, the fibre and needle-shaped structures observed in unirradiated PY65, unirradiated LY, irradiated PY74 and irradiated TiO_2_, might be harmful (Bauer et al. 2020; Boulanger et al*.* 2014b). It is known that the toxicity of fibrous nanoparticles rises as their aspect ratio increases (Lippmann 1990). Aspect-ratio-dependent toxicity is commonly observed in the lungs. Nanofibers approximately 150 nm in thickness and 2, 5, or 10 µm in length are indicative of lung cancer, mesothelioma, and asbestosis, respectively (Boulanger et al*.* 2014a; Lippmann 1990). It has been reported that needle-shaped nanoparticles demonstrate greater toxicity than spherical nanoparticles due to enhanced endocytic processes, higher internalisation rates, and increased adhesiveness to target cell surfaces (Egbuna et al. 2021; Gratton et al*.* 2008).

In contrast, DLS showed an increase in particle size after irradiation of tattoo inks containing TiO_2_ and pigment-TiO_2_ mixtures. This was supported by SEM images which revealed altered morphologies, including large aggregates. When tattoo inks agglomerate rather than decompose during laser therapy, significant toxicity issues may emerge. Initially, if the ink particles aggregate into larger clusters, the body might encounter difficulties to removing them. Larger aggregates are less likely to be transported via the bloodstream or lymphatic system (Champion et al. 2008; Høgsberg et al. 2011), which means that they can stay in the body for extended periods. Over time, these aggregate particles may gradually release hazardous decomposition products into surrounding tissues or the circulation, potentially resulting in localised or systemic poisoning. For example, release of polycyclic aromatic hydrocarbons (PAHs) from black and yellow inks has been reported (Moseman et al. 2024). Prolonged exposure to such carcinogens could increase cancer risk.

Additionally, if the body detects the aggregated particles as foreign, it might trigger a prolonged immune response (Champion and Mitragotri 2006). Larger particles may persist in the skin or lymphatic system, perhaps resulting in chronic local irritation or inflammation. It has been reported that nanoparticle aggregation has the potential to cause inflammatory lung diseases in people (Bantz et al. 2014). In addition, agglomerated carbon nanotubes have been shown to be more hazardous than well-dispersed carbon nanotubes, and longer nanotubes in dimension have been observed to be more toxic (Mohanta et al. 2019; Wick et al. 2007).

TiO₂ nanoparticles have been extensively studied for their toxicity, and their small size and high surface area are often associated with increased reactive oxygen species (ROS) generation, which can lead to oxidative stress and cellular damage (Ma et al. 2012). Studies have shown that nanoparticles exhibit distinct toxicological profiles based on their size, shape, and surface characteristics (Kose et al. 2020; Wang and Fan 2014). Whilst smaller TiO₂ particles have been linked to higher cytotoxicity, the aggregation of TiO₂ into larger clumps, as observed in our study, could modify their interactions with cells or tissues. Larger aggregates may be less efficiently taken up by cells but could contribute to local toxicity through prolonged retention or deposition in tissues, potentially inducing inflammatory responses or cellular damage upon sustained exposure. Additionally, the aggregation of titanium dioxide (TiO₂) nanoparticles on the surface of the BO ink following laser treatment not only alters the surface morphology but may also have implications for the material's toxicity.

Depending on the distribution/elimination of the irradiation products within the body, the cumulative effect of repeated exposure could pose serious health risks to individuals undergoing tattoo removal. More study is needed to examine the in vivo formation of these compounds and fate within the body. Additionally, this study only examined a small number of inks. Further work is required to examine a broader range of inks to comprehensively assess the likely toxicity of tattoo ink irradiation products within the body.

## Conclusion

In conclusion, this study investigated the effects of laser treatment on tattoo inks, specifically focussing on pigments, dried inks, and mixtures of PY14, PY74, and PY65 with TiO_2_. The results revealed a diverse range of degradation products, along with variations in morphology and size. The laser degradation process was significantly influenced by the presence of TiO_2_, with different aggregate morphologies forming when pigment–TiO_2_ mixtures and TiO_2_-containing inks were irradiated. Additionally, the presence of TiO_2_ altered the chemical profile of volatile degradation products detected by GC–MS, along with reducing their concentration, suggesting that TiO_2_ may decrease the extent of degradation. This effect is likely due to TiO_2_ aggregating around pigment particles, thereby limiting the availability of the pigment for degradation. Moreover, the aggregation of TiO_2_ with pigment particles following laser irradiation may impede the efficacy of laser tattoo removal, as the formation of larger particle could reduce their clearance by the body’s natural removal mechanisms. These findings provide insight into the challenges and toxicity associated with the laser removal of tattoos and underscore the need for further research and technological advancements in tattoo removal techniques.

## Supplementary Information

Below is the link to the electronic supplementary material.

Supplementary file1 (DOCX 3249 KB)

## Data Availability

The experimental data supporting this manuscript will be made available upon request.
