# Supplementary material for: Challenges in laser tattoo removal: the impact of titanium dioxide on photodegradation of yellow inks
Source: Arch Toxicol. 2025 Mar 6;99(4):1371–85. doi: 10.1007/s00204-025-03989-2 (PMC11968486; doi:10.1007/s00204-025-03989-2)
Supplement: Supplementary file 1 — Supplementary file1 (DOCX 3249 KB) [file 204_2025_3989_MOESM1_ESM.docx]

**Challenges in Laser Tattoo Removal: The Impact of Titanium Dioxide on Photodegradation of Yellow Inks**

Batool Aljubran^1^, Kirstin Ross^1^, Ula Alexander^1^, Claire E. Lenehan^1*^

^1^ College of Science and Engineering, Flinders University, Sturt Rd, Bedford Park, Adelaide, SA 5042, Australia

* Corresponding author

Author details:

Batool Aljubran – [alju0046@flinders.edu.au](mailto:alju0046@flinders.edu.au), <https://orcid.org/0009-0007-3177-4665>.

Kirstin Ross – [kirstin.ross@flinders.edu.au](mailto:kirstin.ross@flinders.edu.au), <https://orcid.org/0000-0001-5677-9576>.

Ula Alexander - [ula.alexander@flinders.edu.au](mailto:ula.alexander@flinders.edu.au), <https://orcid.org/0000-0002-0864-1289>

Claire Lenehan - [claire.lenehan@flinders.edu.au](mailto:claire.lenehan@flinders.edu.au), <https://orcid.org/0000-0003-3936-8569>

**Supplementary information**

Table SI1: The ingredients of tattoo inks that used in the projects based on the SDS and the label on the bottles.

| Tattoo ink | INTENZE  Lemon Yellow | | INTENZE  Golden Yellow | | INTENZE  Golden Rod | | INTENZE  Bright Orange | |
| --- | --- | --- | --- | --- | --- | --- | --- | --- |
|  | Declaration ingredients | MSDS | Declaration ingredients | MSDS | Declaration ingredients | MSDS | Declaration ingredients | MSDS |
| TiO_2_ | x | x | x | x | - | - | x | x |
| BaSO_4_ | x | x | - | x | - | - | - | x |
| PB15 | x | x | - | - | - | - | - | - |
| PY65 | x | - | - | - | - | - | - | - |
| PY14 | - | x | x | x | x | x | x | x |
| PO13 | - | - | x | - | x | x | x | x |
| Aqua | x | x | x | - | x | x | x | x |
| Glycerine | x | x | x | - | x | x | x | x |
| Hamamelis Virginiana extract | x | x | x | - | x | x | x | x |
| Isopropyl alcohol | - | x | - | - | - | x | - | x |


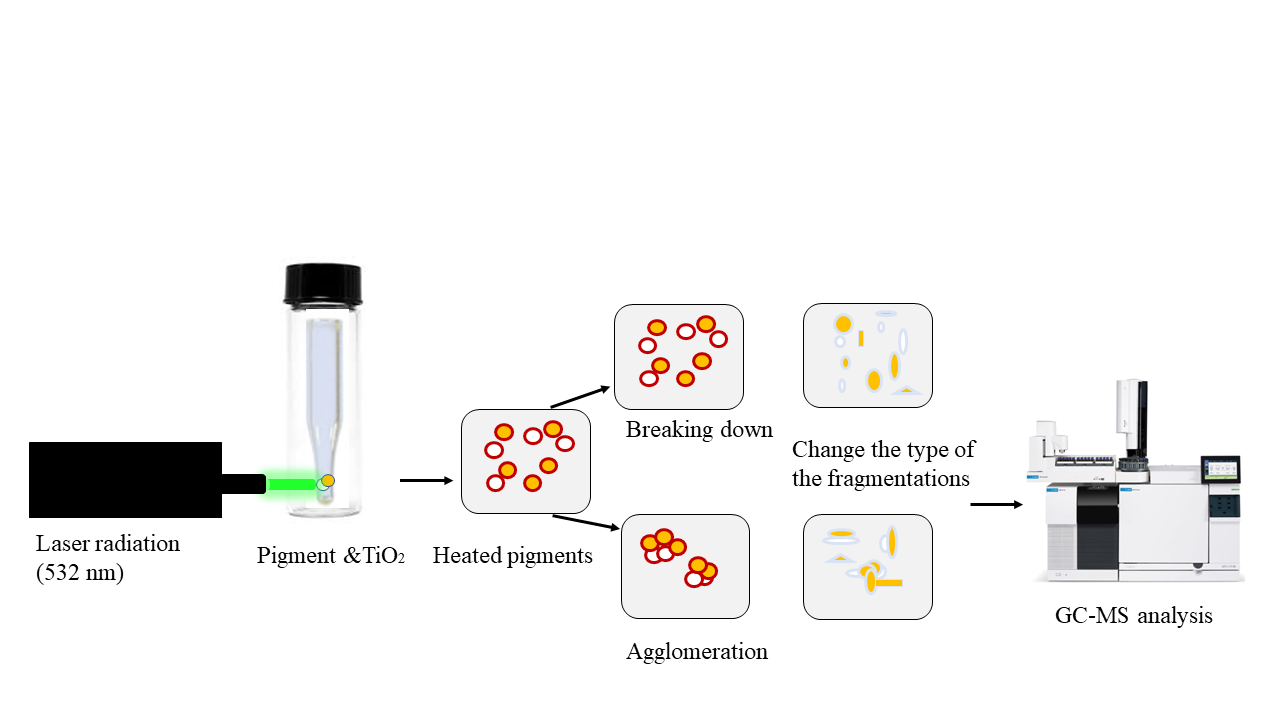


Figure SI1: General methodology of the project (schematic experimental setup).


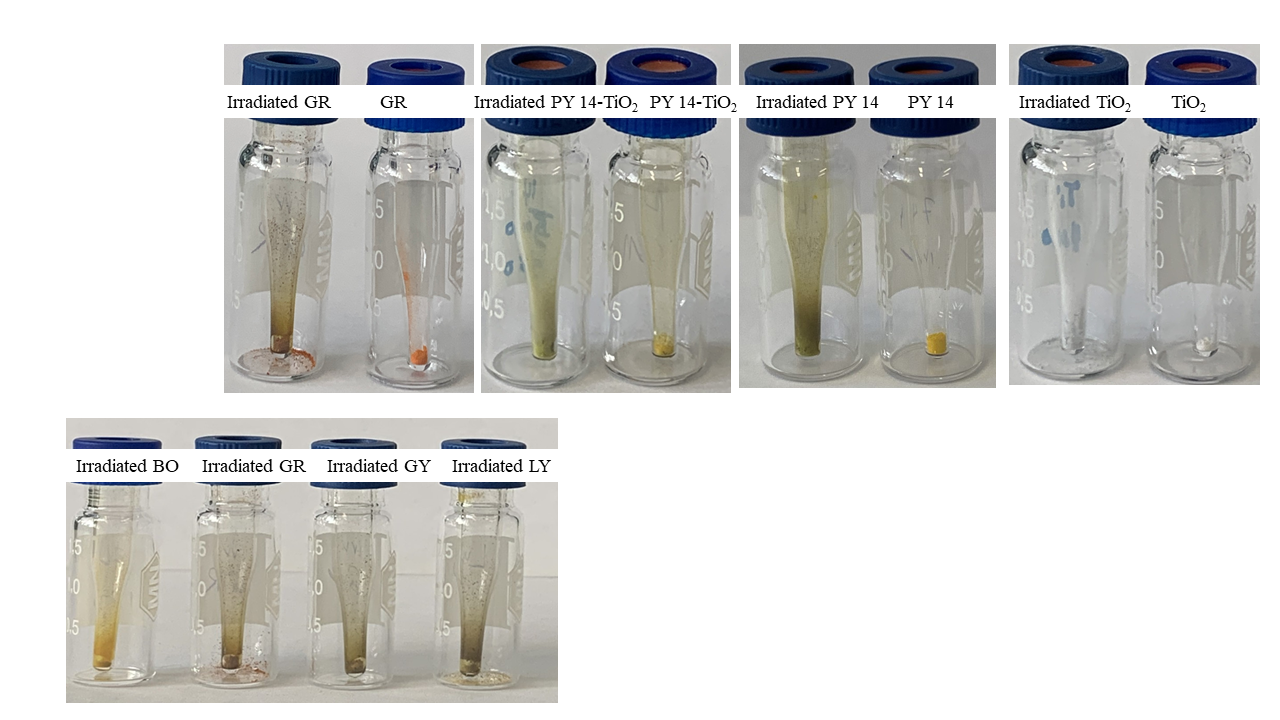


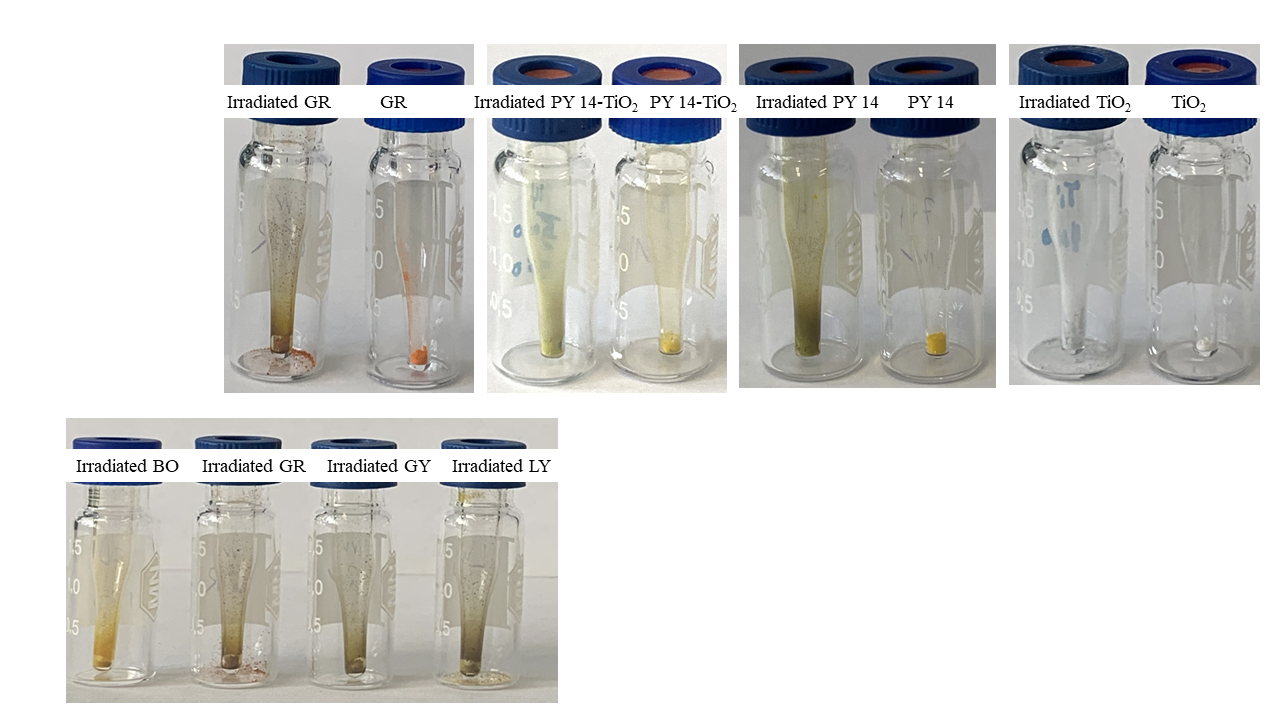


Figure SI2: Photographs of pigments and inks pre- and post-irradiation.

Figure SI3: GC-MS data of unirradiated and irradiated empty vial, pigments, and inks.

Figure SI4: GC-MS spectra of irradiated PY74, and PY65 with and without TiO_2_.


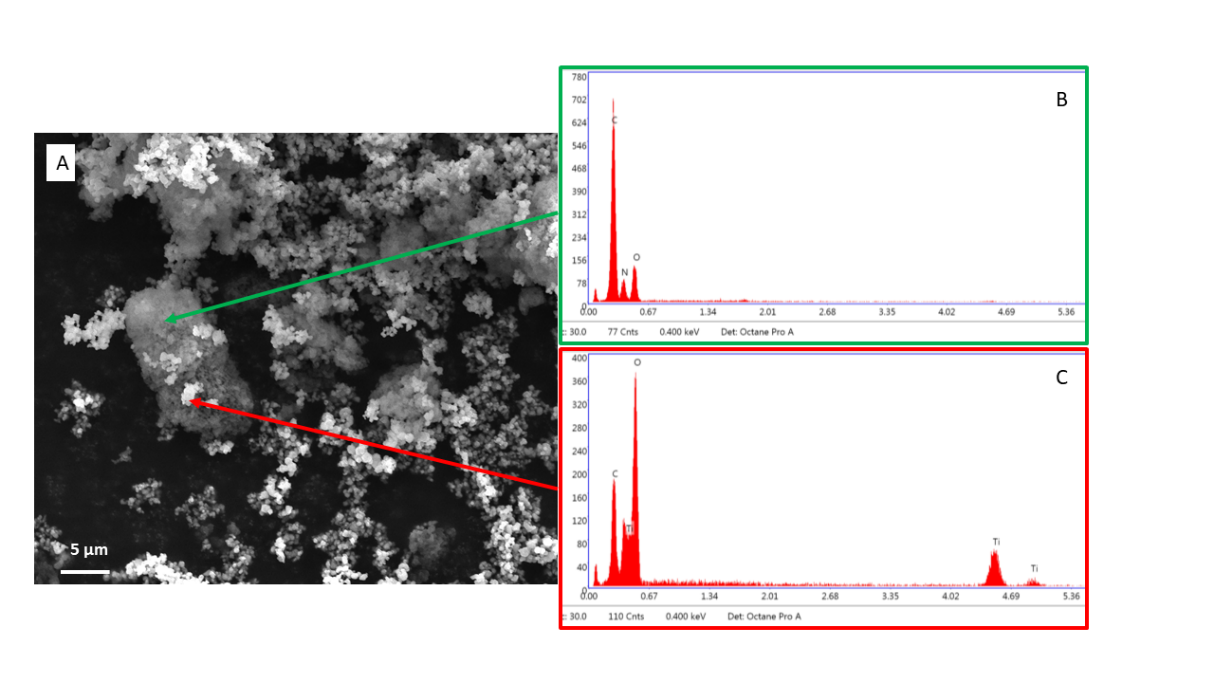


Figure SI5: (A) SEM image if unirradiated mixture of PY74 -TiO_2_, (B, C) EDX scan of the mixture to verify between yellow pigments and TiO_2_ particles.


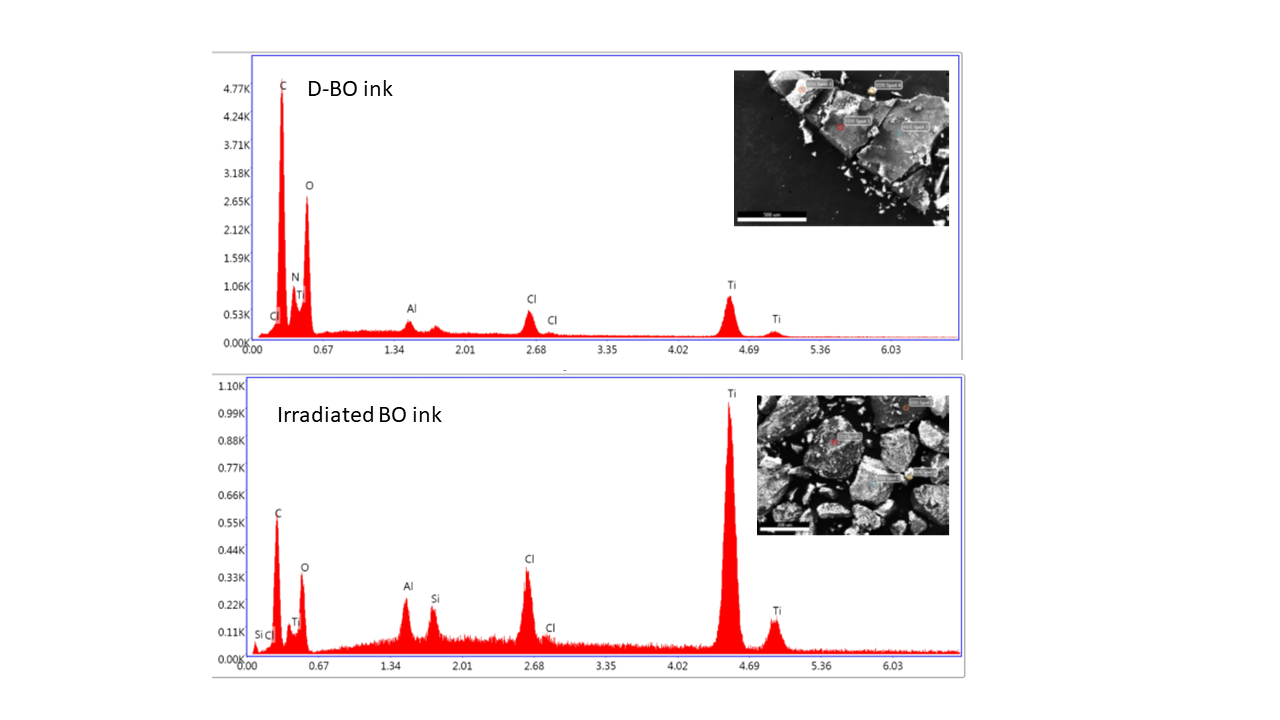


***Figure SI6: EDX analysis surface of unirradiated and irradiated BO ink.***

Figure SI7: DLS measurement of unirradiated pigments and irradiated PY74 and PY65 with and without TiO_2_. Data represents the average value and standard deviation over three repeated. Comparison of the particle size of irradiated pigments with and without TiO_2_, showing the particle size of irradiated pure pigments is smaller than irradiated PY-TiO_2_.

Table SI2: DLS data of unirradiated and irradiated pigments and inks.

| Samples | Particle size (nm) | |
| --- | --- | --- |
|  | Before laser | After laser |
| TiO_2_ | 300 | 473±7 |
| PY14 | 705±39 | 301±20 |
| PY14-TiO_2_ | 531±20 | 461±29 |
| PY74 | 452±46 | 165±21 |
| PY74-TiO_2_ | 396±11 | 334±14 |
| PY65 | 669±24 | 272 ±16 |
| PY65-TiO_2_ | 459±17 | 455±13 |
| LY | 153±16 | 194±49 |
| GY | 149±20 | 492±43 |
| GR | 156±47 | 353±19 |
| BO | 329±20 | 353±27 |

Figure SI8: XRD analysis of unirradiated and irradiated LY, GY, and GR inks. GY ink surface analysis showed high intensity peaks of TiO_2_ after laser irradiation.

Table SI3: The toxicological evaluation and the hazard statement* were made according to ECHA database and Chemwatch.

| Retention time (min) | Compound | Hazard Codes* | Hazard category | Description |
| --- | --- | --- | --- | --- |
| 0.849 | 1,3-butadiyne | H220 | 1A | Extremely flammable gas. |
|  |  | H280 | Compressed gas liquefied gas dissolved gas | Contains gas under pressure may explode if heated |
| 0.920 | 3-butenyl methacrylate | NA | NA | NA |
| 0.953 | 2-propenenitrile | H225 | 2 | Highly Flammable liquid |
|  |  | H301 | 3 | Toxic if swallowed |
|  |  | H311 | 3 | Toxic in contact with skin |
|  |  | H315 | 2 | Causes skin irritation |
|  |  | H317 | 1, 1A, 1B | May cause an allergic skin reaction |
|  |  | H319 | 2/2A | Causes serious eye damage |
|  |  | H331 | 3 | Toxic if inhaled |
|  |  | H335 | 3 | May cause respiratory irritation |
|  |  | H350 | 1, 1A, 1B | May cause cancer |
|  |  | H361 | 2 | Suspected of damaging fertility or the unborn child |
|  |  | H411 | 2 | Toxic to aquatic life with long lasting effects |
| 1.144 | benzamide | H302 | 4 | Harmful if swallowed. |
|  |  | H341 | 2 | Suspected of causing genetic defects. |
|  | benzonitrile | H227 | 4 | Combustible liquid. |
|  |  | H302 | 4 | Harmful if swallowed. |
|  |  | H312 | 4 | Harmful in contact with skin. |
| 1.397 | benzene | H225 | 2 | Highly flammable liquid and vapour. |
|  |  | H304 | 1 | May be fatal if swallowed and enters airways. |
|  |  | H315 | 2 | Causes skin irritation. |
|  |  | H319 | 2/2A | Causes serious eye irritation. |
|  |  | H336 | 3 | May cause drowsiness or dizziness. |
|  |  | H340 | 1, 1A, 1B | May cause genetic defects. |
|  |  | H350 | 1, 1A, 1B | May cause cancer. |
|  |  | H360Fd | 1, 1A, 1B | May damage fertility |
|  |  | H372 | 1 | Causes damage to organs through prolonged or repeated exposure. Suspected of damaging the unborn child. |
|  |  | H401 | 2 | Toxic to aquatic life. |
| 1.635 | 2-propenoic acid-ethyl ester | NA | NA | NA |
| 1.702 | N, N’- dimethyl-1,2 bis(aminooxy)ethane | NA | NA | NA |
| 1.70 | methyl 1-dideutreio-2-propenyl ether | NA | NA | NA |
| 1.722 | methyl methacrylate | H225 | 2 | Highly flammable liquid and vapour. |
|  |  | H315 | 2 | Causes skin irritation. |
|  |  | H317 | 1, 1A, 1B | May cause an allergic skin reaction. |
|  |  | H319 | 2/2A | Causes serious eye irritation. |
|  |  | H335 | 3 | May cause respiratory irritation. |
|  |  | H336 | 3 | May cause drowsiness or dizziness. |
| 2.412 | toluene | H225 | 2 | Highly flammable liquid and vapour. |
|  |  | H302 | 4 | Harmful if swallowed. |
|  |  | H304 | 1 | May be fatal if swallowed and enters airways. |
|  |  | H315 | 2 | Causes skin irritation. |
|  |  | H319 | 2/2A | Causes serious eye irritation. |
|  |  | H336 | 3 | May cause drowsiness or dizziness. |
|  |  | H361d | 2 | Suspected of damaging the unborn child. |
|  |  | H373 | 2 | May cause damage to organs through prolonged or repeated exposure. |
| 5.701 | styrene | H226 | 3 | Flammable liquid and vapour. |
|  |  | H302 | 4 | Harmful if swallowed. |
|  |  | H315 | 2 | Causes skin irritation. |
|  |  | H319 | 2/2A | Causes serious eye irritation. |
|  |  | H332 | 4 | Harmful if inhaled. |
|  |  | H351 | 2 | Suspected of causing cancer. |
|  |  | H361d | 22 | Suspected of damaging the unborn child. |
|  |  | H373 | 2 | May cause damage to organs through prolonged or repeated exposure. |
| 7.208 | benzene methanol | H302 + H332 | 4 | Harmful if swallowed or if inhaled. |
|  |  | H319 | 2/2A | Causes serious eye irritation. |

*Association between hazards code and toxicity of fragments produced by laser treatment of inks was according to globally harmonized system of classification and labelling of chemicals (GHS*).*[*http://www.unece.org/fileadmin/DAM/trans/danger/publi/ghs/ghs_rev08/ST-SG-AC10-30-Rev8e.pdf*](http://www.unece.org/fileadmin/DAM/trans/danger/publi/ghs/ghs_rev08/ST-SG-AC10-30-Rev8e.pdf)*.*
